# Profiling and Role of Bioactive Molecules from *Puntius sophore* (Freshwater/Brackish Fish) Skin Mucus with Its Potent Antibacterial, Antiadhesion, and Antibiofilm Activities

**DOI:** 10.3390/biom10060920

**Published:** 2020-06-17

**Authors:** Mitesh Patel, Mohammad Saquib Ashraf, Arif Jamal Siddiqui, Syed Amir Ashraf, Manojkumar Sachidanandan, Mejdi Snoussi, Mohd Adnan, Sibte Hadi

**Affiliations:** 1 Bapalal Vaidya Botanical Research Centre, Department of Biosciences, Veer Narmad South Gujarat University, Surat, Gujarat 395007, India; patelmeet15@gmail.com; 2 Department of Clinical Laboratory Sciences, College of Applied Medical Science, Shaqra University, Al Dawadimi 17472, Saudi Arabia; ashrafsaquib@gmail.com; 3 Department of Biology, College of Science, University of Hail, P.O. Box 2440, Hail, Saudi Arabia; arifjamal13@gmail.com (A.J.S.); snmejdi@yahoo.fr (M.S.); 4 Department of Clinical Nutrition, College of Applied Medial Sciences, University of Hail, P.O. Box 2440, Hail, Saudi Arabia; amirashrafy2007@gmail.com; 5 Department of Oral Radiology, College of Dentistry, University of Hail, P.O. Box 2440, Hail, Saudi Arabia; smanojk68@gmail.com; 6 Laboratory of Bioresources: Integrative Biology and Valorization, (LR14-ES06), University of Monastir, Higher Institute of Biotechnology of Monastir, Avenue Tahar Haddad, BP 74, Monastir 5000, Tunisia; 7 School of Forensic and Applied Sciences, University of Central Lancashire, Preston PR1 2HE, UK

**Keywords:** *Puntius sophore*, antibiofilm, antiadhesion, antibacterial, biofilms, bioactive molecules, exopolysaccharide, antimicrobial peptides, HR-LCMS

## Abstract

Epidermal fish mucus comprises of diverse bioactive metabolites which plays an immense role in defense mechanisms and other important cellular activities. Primarily, this study aims to screen the unexplored mucus extract of *Puntius sophore* (*P. sophore*) for its antagonistic potential against common pathogens, which are commonly implicated in foodborne and healthcare associated infections, with effects on their adhesion and biofilm formation. Profiling of the skin mucus was carried out by High Resolution-Liquid Chromatography Mass Spectrometry (HR-LCMS), followed by antibacterial activity and assessment of antibiofilm potency and efficacy on the development, formation, and texture of biofilms. Furthermore, bacterial cell damage, viability within the biofilm, checkerboard test, and cytotoxicity were also evaluated. As a result, *P. sophore* mucus extract was found to be effective against all tested strains. It also impedes the architecture of biofilm matrix by affecting the viability and integrity of bacterial cells within biofilms and reducing the total exopolysaccharide content. A synergy was observed between *P. sophore* mucus extract and gentamicin for *Escherichia coli* (*E. coli*), *Pseudomonas aeruginosa* (*P. aeruginosa*), and *Bacillus subtilis* (*B. subtilis*), whereas, an additive effect for *Staphylococcus aureus* (*S. aureus*). Thus, our findings represent the potent bioactivities of *P. sophore* mucus extract for the first time, which could be explored further as an alternative to antibiotics or chemically synthesized antibiofilm agents.

## 1. Introduction

Biofilms are colossally structured, densely packed with surface affixed population of single or multiple microbial cells in autogenic extracellular polysaccharide matrix. They are comprised of diverse proteins, lipids, polysaccharides, nucleic acids, and other chemical or biochemical constituents [1,2,3,4]. More than 90% of bacteria can exist in biofilm state and can swiftly disperse into a variety of environmental sites, including the human body. Bacteria within biofilms displays an exceedingly exalted pattern of adaptive resistance to antibiotics and other bactericides in contrast to their planktonic form [5,6,7]. They are also completely resistant to host immune defenses, antibiotic therapies, and various physicochemical factors like heavy metals, salinity, acidity, ultraviolet light, and phagocytosis. Therefore, removal of biofilms becomes strenuous, once established [8,9]. This adaptive antibiotic resistance mechanism of bacteria in biofilms also act as a hurdle in the treatments of biofilm related acute and chronic diseases like bacterial vaginosis, nosocomial pneumonia, urinary tract infections, surgical wound infections, catheter infections, burn wound infections, middle ear infections, gingivitis, etc. [10]. Due to this reason, biofilm formation is not only a problem to health care sector, but it is a major global challenge, imposing serious complications to other sectors including oceanic, dairy, aquaculture, food and beverage industries, etc. [11]. Therefore, there is an urgent need to develop/explore novel and natural biologically active molecules to control biofilms rather than with antibiotics or other chemically synthesized agents.

All through the time of drug evolution, nature has always been the foremost origin for the discovery of novel bioactive compounds/medications, essential for fighting against infections and various diseases [11,12]. Over the past decade, novel perspectives in impeding biofilm formation have been extensively developed from natural products, especially from plants, as they demonstrated antimicrobial and chemo-preventive properties [13,14]. Recently, fishes have been also considered as an unexploited source of prospective novel pharmaceutical products, nutraceuticals, functional foods, and therapeutics [15]. They display numerous structural characteristics with plentiful sources of bioactive compounds, which could be utilized as novel and potent antimicrobial and antibiofilm drugs. Though fishes are a known enormous source of bioactive compounds, very few fishes have been tested for their biological applications, specifically for their antibiofilm potency. In this context, we evaluated the antibiofilm and antibacterial potential of *P. sophore* (F. Hamilton, 1822) mucus extract.

*Puntius sophore (P. sophore*), commonly known as pool barb, spotfin swamp barb, ‘PhabouNga’, or stigma barb is a freshwater cyprinid fish widely distributed in Asia (India, Nepal, Bangladesh, Myanmar, Bhutan, Afghanistan, Pakistan, and China). It is one of the nutritionally superior small indigenous fish known to be rich in nutrients, proteins, unsaturated fatty acid, and vitamins [16]. In rural communities, it is a prime food and a pivotal source of micronutrients essential in preventing malnutrition, vitamin and mineral deficiencies [17]. Very popular and important traditional fermented fish products Shidol’ and ‘Ngari’ are prepared from *P. sophore.* Extracts of this fish have been reported to exert an antioxidant potential [18]. However, as per our knowledge, no study has been reported detailing the antimicrobial, antiadhesion, and antibiofilm effects of the *P. sophore* skin mucus extract to date. Thus, the aim of this study was to explore the antagonistic potency of *P. sophore* mucus extract against planktonic and biofilm producing pathogenic bacteria using different *in vitro* approaches.

## 2. Materials and Methods 

### 2.1. Ethics Statement

*Puntius sophore (P. sophore*) was only used to collect the mucus from skin, and collection was carried out in accordance with the ethical guidelines and were strictly adhered to while maintaining and handling the fish. *P. sophore* was not harmed or killed during/for any experiment throughout this research.

### 2.2. Strains, Materials, and Growth Conditions

The strains used in this study were two Gram-positive bacterial strains: *B. subtilis* (MTCC 121), *S. aureus* (MTCC 96) and two Gram-negative bacterial strains *E. coli* (MTCC 9537) and *P. aeruginosa* (MTCC 741) [19,20]. All bacterial strains were obtained from the Microbial Type Culture Collection (MTCC), Chandigarh, India and maintained on Muller-Hinton Agar (MHA) before each experiment. Pure bacterial cultures were prepared by transferring a single colony into a fresh medium and grown overnight at 37 °C. The 0.5 Mc Farland standard (10^8^ CFU/mL) was matched by adjusting the turbidity of the culture with sterile saline solution. Biofilms of all bacterial strains were formed on 96-well microtiter plates, filled with 100 µL Muller-Hinton Broth (MHB), 1% glucose, and cells (10^7^ cells/mL) for 24 h at 37 °C. For positive control, gentamicin standard antibiotic was used throughout. 

### 2.3. Collection and Maintenance of Fish

Growing live *P. sophore* were collected from the natural water bodies and transferred to the laboratory (Surat, India). A total of 20 fish were maintained in a 1000 L of fish tank at a water temperature 27 ± 2 °C and pH of 7 ± 2. The total length of the fish ranged from 8.3 to 12.10 cm and total body weight ranged from 14.32 to 20.68 g. Half of the water in tank was changed on alternate days to retain hygiene conditions. They were daily monitored for their health, as only healthy fish were sampled for mucus collection and fish with any lesions were taken out from the tank immediately. They were fed every day with the prepared feed of wheat flour, rice bran, groundnut oil cake, and mixture of minerals at 4% of their body weight during the acclimation period. 

### 2.4. Collection of Fish Mucus

After seven days of acclimation in laboratory conditions, fish were starved for one day and washed with 2% of potassium permanganate before collection of mucus. Mucus sample was collected with the help of a sterile spatula by softly scraping from dorsal side in anterior to posterior direction, from head to tail, at regular intervals in a day. No anesthesia or chemical was used. Collected mucus sample was centrifuged at 8000 rpm for 10 min to remove precipitates present in the sample. The supernatant was collected, and acidic extract of mucus was prepared according to Diamond et al. with slight modifications [21]. To prepare acidic extract, 50 mL of pooled mucus sample was mixed with 50 mL of 10% acetic acid and boiled for 5 min in boiling water bath. The mixture was then centrifuged at 10,000 rpm for 30 min at 4 °C. The supernatant was collected and lyophilized. The final dried extract was resuspended in deionized water to make 2000 µg/mL concentration. Prepared mucus aqueous extract was stored at 0 °C for further use.

### 2.5. Antibacterial Activity

Antibacterial capability of *P. sophore* mucus extract was evaluated by agar cup/well diffusion method. All bacterial strains were uniformly (1000 µL) spread over the plates and wells were punctured with the help of gel puncture. Into each respective well, 100 µL of mucus extract (2000 µg/mL) was inoculated and plates were incubated at 37 °C for 24 h. On the next day, zones of inhibition were calculated. For positive control, gentamicin standard antibiotic was used.

### 2.6. Effect of Puntius sophore Mucus Extract on Growth Kinetics of Bacteria

The effect of *P. sophore* mucus extract on the growth kinetics of bacteria was observed by inoculating 0.5 mL of all grown bacterial strains individually into 150 mL of sterile nutrient broth containing 1 mL of mucus extract (2000 µg/mL). A flask without mucus extract and having only culture served as the control. Later, growth kinetics were measured for each bacterial strain by taking absorbance at 600 nm at each 1 h time interval.

### 2.7. Determination of Minimum Inhibitory Concentration (MIC) by Serial Dilution Assay

The MIC of mucus extract was carried out via microdilution methods using MHB as described by Clinical and Laboratory Standards Institute (CLSI) with slight modifications [22]. Bacterial inoculums were prepared in MHB at 37 °C for 24 h. The mucus extract was two-fold diluted ranging from 2000 to 0.48 µg/mL (80 µL as final volume) with final phosphate buffer saline concentration <1%. Afterwards, 20 µL of bacterial suspensions (10^8^ CFU/mL) and 100 µL of MHB were loaded onto microtiter plates and the test was accomplished in 200 µL of final volume. The absorbance of each well was determined using Epoch^TM^ microplate spectrophotometer at 600 nm. Plates were then incubated at 37 °C for 24 h. After incubation, the absorbance was read again in the reader at the same wavelength and the obtained absorbance values were subtracted from those obtained before incubation. Assessment was carried out simultaneously for bacterial growth control (MHB + bacteria + mucus extract vehicle) and sterility control (MHB + mucus extract vehicle), as well as for the positive control gentamicin was used. MICs were recorded as the lowest concentration that inhibits the bacterial growth [23]. 

### 2.8. Determination of Minimum Bactericidal Concentration (MBC)

MBC was characterized following the MIC assay by spreading 5 µL of sample on MHA plates from the wells that exhibited no evident growth. Plates were then incubated at 37 °C for 18–24 h. MBC was then recorded, at the lowest concentration that yielded three or fewer colonies i.e., 99% of the inoculum was killed [24].

### 2.9. Determination of Fractional Inhibitory Concentration Index (FICI)

Microdilution checkerboard test was used for determining the FICI of antibacterial combination of *P. sophore* mucus extract and gentamicin [25]. Then, 96-well microtiter plates with MHB, *P. sophore* mucus extract and gentamicin in two-fold serial concentrations were used for the assay. Cell suspensions (100 μL) of respective bacterial strains, *P. sophore* mucus extract (100 μL) and gentamicin (100 μL) were incubated at 37 °C for 24 h. FICI for the combination was assessed [26] as:

FICI = FIC of Drug A + FIC of Drug B

where, FIC A is the MIC of Drug A in the combination/MIC of Drug A alone. FIC B is the MIC of Drug B in the combination/MIC of Drug B alone. The amalgamation is believed to be synergistic; when, FICI is <0.5. The amalgamation is believed to be additive; when, the FICI is >0.5 to <2. The amalgamation is believed to be antagonistic; when, the FICI is >2.

### 2.10. Biofilm Assay

Static biofilm formation was assayed in 96-well polystyrene plates by crystal violet method as described by Lee et al. [27] with some modifications. Briefly, overnight culture of respective bacterial strains together with MHB (200 µL) at an initial turbidity of 0.05 at 600 nm and incubated at 37 °C without shaking for 24 h. After the period of incubation, planktonic cells were removed by washing three times with phosphate buffered saline (PBS), dried, and stained with 0.1% crystal violet for 20 min. Surplus dye was taken out, dissolved in 95% ethanol, and absorbances were measured at 570 nm.

### 2.11. Assessment on Established Biofilms 

The effect of *P. sophore* mucus extract on biofilms was performed by established method [28]. Biofilms of all bacterial strains were formed on 96-well microtiter plates, filled with MHB, 1% glucose, and cells (10^7^ cells/mL) for 24 h at 37 °C. After the period of incubation, planktonic cells were gently discarded, and the wells were washed three times with PBS. Then, *P. sophore* mucus extract (MIC) (200 µL) was added into the wells and kept for further incubation at 37 °C for 24 h. Absorbance was read at 492 nm at 0 and after 24 h. All assays were performed in triplicate. MHB medium with individual bacterial strain was used as biofilm growth control. The percentage of biofilm inhibition was estimated as follows (Equation (1)):

[(OD (control) − OD (test)/OD control)] × 100.(1)

where, OD: Optical Density.

### 2.12. Assessment on Adherence of Biofilms

The effect of *P. sophore* mucus extract to inhibit biofilm formation was accomplished by spectrophotometric method as stated [29] in 96-well microtiter plates. Cell suspensions (100 µL) of respective bacterial strains (10^8^ CFU/mL) and *P. sophore* mucus extract (MIC) were incubated at 37 °C for 24 h. After the incubation, planktonic cells were removed by washing the wells very delicately with PBS (200 µL). Biofilms developed by adherent cells were stained with 0.1% crystal violet (100 µL), followed by incubation at 37 °C for 30 min. PBS was used to wash off the extra stain and plates were then fixed with 95% ethanol (200 µL), followed by further incubation at 37 °C for 15 min. Absorbance was read spectrophotometrically at 590 nm. The percentage inhibition was estimated as follows (Equation (2)):

[(OD (control) − OD (test)/OD control)] × 100(2)

where, OD: Optical Density

### 2.13. Assessment of Antibiofilm Activity by Light Microscopy (LM)

Light microscopic assessment of all bacterial biofilms was accomplished following the prescribed method [30] with some modifications. Overnight grown culture of all bacterial strains was added to a 5 mL freshly prepared MHB with 1% glucose. Then, 500 µL of inoculated broth (10^8^ CFU/mL) was transferred to 24-well microtiter plates containing 1 × 1 cm size cover slip. Treatment was carried out with 500 µL of the *P. sophore* mucus extract (final concentration = MIC). Gentamicin and sterile water in the same amount were used as positive and negative control, respectively. Biofilms on glass cover slips after incubation in static condition for 24 h at 37 °C were removed gently and washed with PBS, followed by staining with 0.1% crystal violet. Excess stain was washed off using de-ionized water and allowed to air dry for 5 min. Stained cover slips were observed under LM (Axioscope A1, ZEISS, Oberkochen, Germany).

### 2.14. Assessment of Antibiofilm Activity by Fluorescence Microscopy (FM)

The biofilms of all bacterial strains were allowed to form on 1 × 1 cm size cover slip with all respective treatments as stated above. Biofilms formed on coverslips were stained with 1% acridine orange. Excess stain was drained off, followed by washing with de-ionized water and allowed to air dry for 5 min. Then, the stained cover slips were visualized under FM (Axioscope A1, ZEISS).

### 2.15. Assessment of Antibiofilm Activity by Scanning Electron Microscopy (SEM)

All bacterial biofilms were analyzed by SEM (in the presence and absence of the *P. sophore* mucus extract with controls against respective strains as stated above). First, 2.5% glutaraldehyde was used for fixing the biofilms on glass coverslips for 30 min at 37 °C. The fixed samples were then washed down three times with PBS and dehydrated through a graded series of 30%, 50%, 70%, 90%, and 100% of ethanol solutions for 15 min in each. Then, ethanol was reinstated with isoamyl acetate and the samples were freeze dried. Coverslips were mounted on aluminum holder, with gold coating using E-1010 ion sputter (Hitachi^®^, Tokyo, Japan) followed by observation under SEM (S-34002N SEM, Hitachi^®^) [31,32].

### 2.16. Biofilm Metabolic Activity—XTT Reduction Assay 

The colorimetric 2, 3-Bis(2-methoxy-4-nitro-5-sulfophenyl)-5-[(phenyl-amino)carbonyl]-2H-tetrazolium hydroxide (XTT) reduction test was carried out to estimate the bacterial cells viability within the biofilms by following previously reported methods [33,34,35]. Overnight culture of respective bacterial strains was inoculated into MHB (200 µL) at an initial turbidity of 0.1 at 600 nm, grown with and without mucus extract at 37 °C without shaking for 24 h. After incubation, plates were washed three times with distilled water to remove the planktonic cells and wells were filled with sterile PBS (100 µL) and freshly prepared solution of XTT-menadione (100 µL). The plate was then incubated for 5 h at 37 °C in the dark, followed by transferring of colored supernatant (100 µL) from each well into a new 96-well microtiter plate. Using a microplate reader, absorbance was then measured at 480 nm. The percentage of surviving bacterial population was calculated as follows (Equation (3)):

[(OD (fish mucus treated sample) − OD (negative control)/OD of untreated control)] × 100(3)

where, OD: Optical Density

### 2.17. Cell Damage Assay

To evaluate the bacterial cell damage within the biofilms, lactate dehydrogenase (LDH) assay was performed. Briefly, culture of respective bacterial strains (100 µL) with MHB (100 µL) was added into 96-well microtiter plates and incubated at 37 °C without shaking for 24 h. After incubation, planktonic cells were removed by washing three times with sterile PBS. Mucus extract (MIC) (100 µL) was then added and further incubated at 37 °C without shaking for 24 h. At the end of incubation, supernatant was collected and used for the estimation of LDH activity via LDH assay kit (Sigma^®^, Bangalore, India) at 480 nm. MHB and bacterial culture was used as a negative control. 

### 2.18. Extracellular Polysaccharide (EPS) Production Assay

Ruthenium red staining assay was used for determining the effect of *P. sophore* mucus extract in reducing the EPS matrix production in all tested bacterial strains biofilm [36]. Cell suspensions (100 μL) of respective bacterial strains (10^8^ CFU/mL) and mucus extract (MIC) were incubated at 37 °C for 24 h. After the incubation, planktonic cells were removed by washing the wells very delicately with phosphate buffered saline (PBS) (200 μL). Biofilms developed by adherent cells were stained with 0.01% ruthenium red (Sigma Aldrich^®^) (200 μL) to each well. Ruthenium red (200 μL) was used to fill the wells without biofilms, and served as blank, followed by incubation at 37 °C for 60 min. Afterwards, the liquid holding the residual stain was resettled in a new microtiter plate and the absorbance was read at 450 nm. Quantity of the dye fixed to biofilms was calculated as follows (Equation (4)):

Abs_BF_ = Abs_B_ − Abs_S_ (4)

where, Abs_B_ = absorbance of blanks, Abs_S_ = absorbance of residual stain collected from sample wells.

### 2.19. Cytotoxicity Assay

Human normal colon cells (CRL-1831) were cultured in DMEM medium (Hi-Media^®^, Mumbai, India) supplemented with 5% FBS, 1% penicillin-streptomycin at 37 °C in a humidified atmosphere of 5% CO_2_/95% air. The culture medium was replaced every 2–3 days. Cytotoxic effect of *P. sophore* mucus extract was determined by the MTT [3-(4,5-dimethylthiazol-2-yl)-2,5-diphenyltetrazolium bromide] assay. Cells were seeded in 96-well plates at a density of more than 1 × 10^5^ cells per well and incubated in humidified atmosphere containing 5% CO_2_ at 37 °C up to adherence. Cells were then treated with different concentrations of *P. sophore* mucus extract (20–100 μg/mL) for 48 h. After incubation, cells were washed with PBS solution and subjected to 100 μL of MTT solution (5 mg/mL) and further incubated for 4 h. Finally, the medium was removed and 100 μL of dimethyl sulfoxide (DMSO) was added to solubilize the formazan crystals. Amount of formazan crystal was determined by measuring the absorbance at 570 nm using enzyme-linked immunosorbent assay (ELISA) reader. Assays were done in triplicate and viability was expressed in % of control.

### 2.20. Identification and Analysis of Bioactive Metabolites by High Resolution-Liquid Chromatography Mass Spectroscopy (HR-LCMS)

Biochemical metabolites present in *P. sophore* mucus extract was carried out using Ultra High-Performance Liquid Chromatography with Photodiode Array (UHPLC-PDA)-Detector Mass Spectrophotometer (HR-LCMS 1290 Infinity UHPLC System), Agilent Technologies^®^, Santa Clara, California, USA. The liquid chromatographic system consisted of a HiP sampler, binary gradient solvent pump, column compartment and quadrupole time of flight mass spectrometer (MS Q-TOF) with dual Agilent Jet Stream Electrospray (AJS ES) ion source. First, 10 µL of sample was injected into the system, followed by separation in SB-C18 column (2.1 × 50 mm, 1.8 µm particle size). Then, 1% formic acid in deionized water (solvent A) and acetonitrile (solvent B) were used as solvents. Flow rate of 0.350 mL/min was used, while, MS detection was performed in MS Q-TOF. Metabolites were identified via their mass spectra and their unique mass fragmentation patterns [37]. 

### 2.21. Statistical Analysis

All experiments were carried out in triplicate. The results are presented as mean values and error bars represent standard error of mean (SEM) of results from three replicate experiments. Statistical analysis was performed using GraphPad Prism 5.0 Software and significance was determined using Student’s *t*-test. *p* values < 0.05 were considered significant. 

## 3. Results

### 3.1. Antibacterial Susceptibility Profile of Puntius sophore Mucus Extract

**Figure 1:**
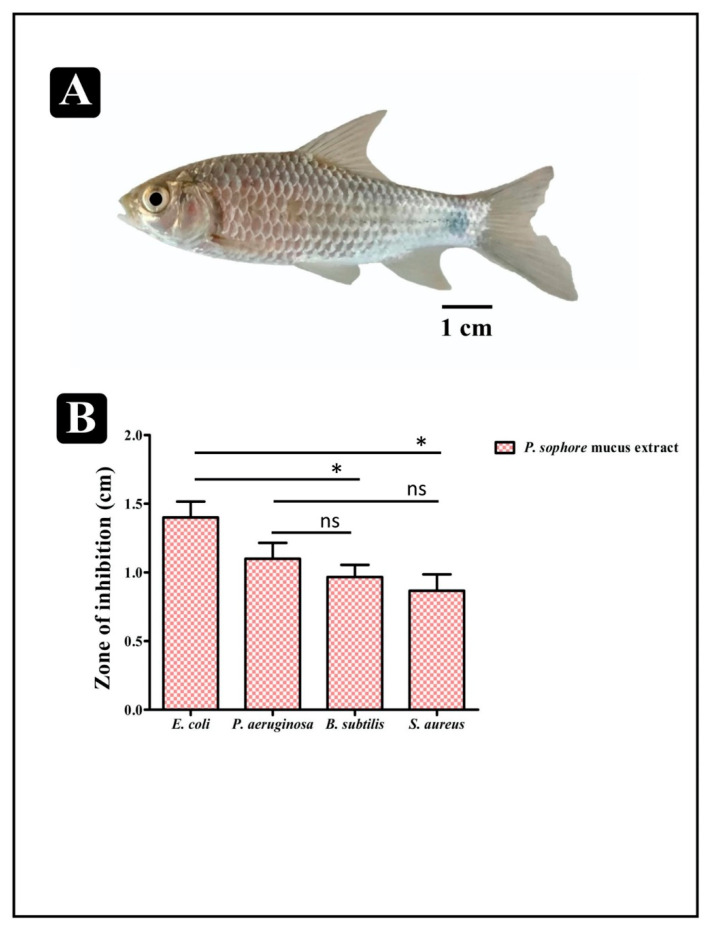
*Puntius sophore* and its antibacterial activity. (**A**) *Puntius sophore* (**B**) antibacterial activity against *E. coli*, *P. aeruginosa, B. subtilis*, and *S. aureus*. All experiments were carried out in triplicate, and data represent the mean ± SD. Statistical significance between different groups was determined using Student’s *t*-test (* *p* < 0.05).

**Figure 2:**
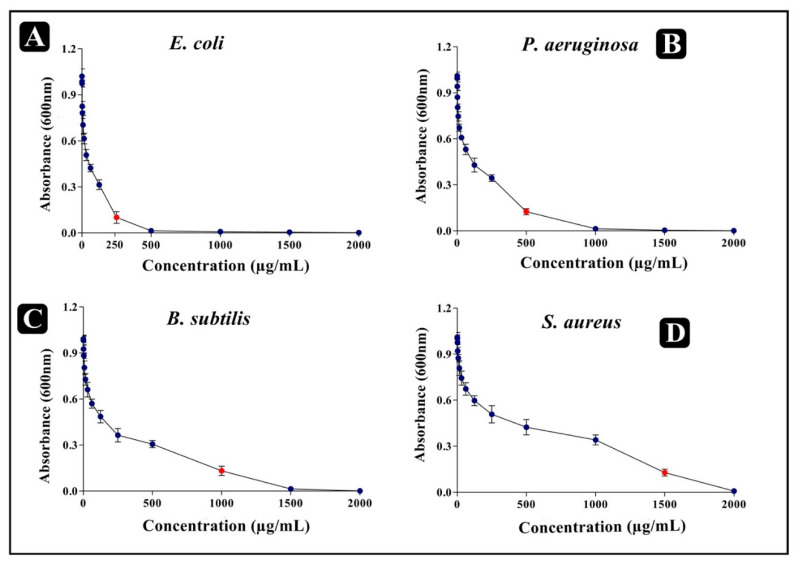
Determination of MIC after taking optical density at 600 nm (**A**) *E. coli*, (**B**) *P. aeruginosa*, (**C**) *B. subtilis*, and (**D**) *S. aureus.* All experiments were carried out in triplicate, and data represent the mean ± SEM.

The antibacterial activity of the mucus extract of *P. sophore* was evaluated against Gram-positive (*B. subtilis* and *S. aureus*) and Gram-negative (*P. aeruginosa* and *E. coli*) bacteria using agar cup/well diffusion method. Results of antibacterial activity are presented in the form of zone of inhibition and revealed substantial antagonistic activity against all the four tested bacterial strains. *E. coli* and *P. aeruginosa* were found to be more susceptible when compared to *B. subtilis* and *S*. *aureus* (Figure 1B). MICs values were ranged from 250 to 1500 µg/mL (Figure 2) and MBC values were shown to be 2–3 times higher than the MIC values (Table 1). These results advocate that the mucus extract of *P. sophore* exhibited inhibitory activities against all tested pathogens. 

### 3.2. Bacterial Killing Assay

**Figure 3:**
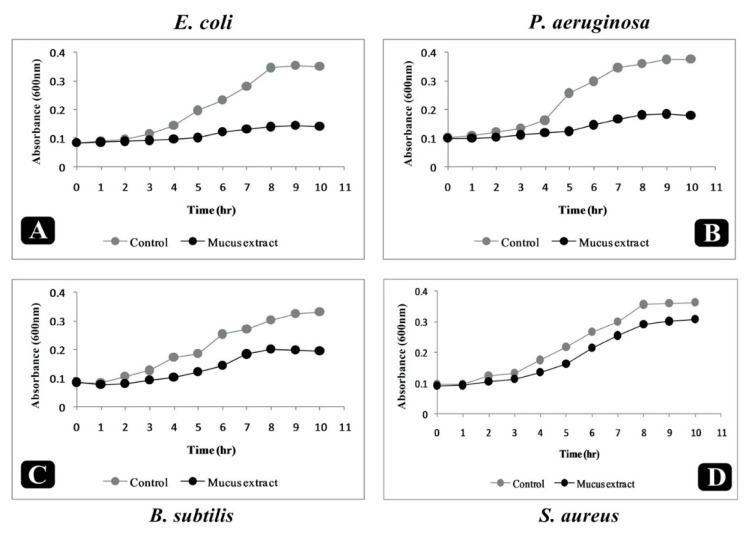
Growth kinetics analysis of bacteria. (**A**) Growth kinetics of *E. coli*, with and without mucus extract, (**B**) growth kinetics of *P. aeruginosa*, with and without mucus extract, (**C**) growth kinetics of *B. subtilis*, with and without mucus extract, and (**D**) growth kinetics of *S. aureus*, with and without mucus extract.

To evaluate the inhibition effect of *P. sophore* mucus extract on pathogenic bacteria, a growth kinetics assay was carried out in presence of mucus extract. Results of growth kinetics analysis displayed the efficacious inhibition of all tested bacterial strains. In contrast to control, growth of all bacterial strains demonstrated a delayed lag phase and protracted logarithmic phase (Figure 3A–D).

### 3.3. Checkerboard Test

For both *P. sophore* mucus extract and gentamicin, the checkerboard assay showed a decline in the MIC values. This clearly suggests a plausible interaction between each other and exhibited a significant result of synergistic action between both *P. sophore* mucus extract and gentamicin for all tested organisms except *S. aureus* (Table 2).

### 3.4. Antibiofilm Properties of P. sophore Mucus Extract

**Figure 4:**
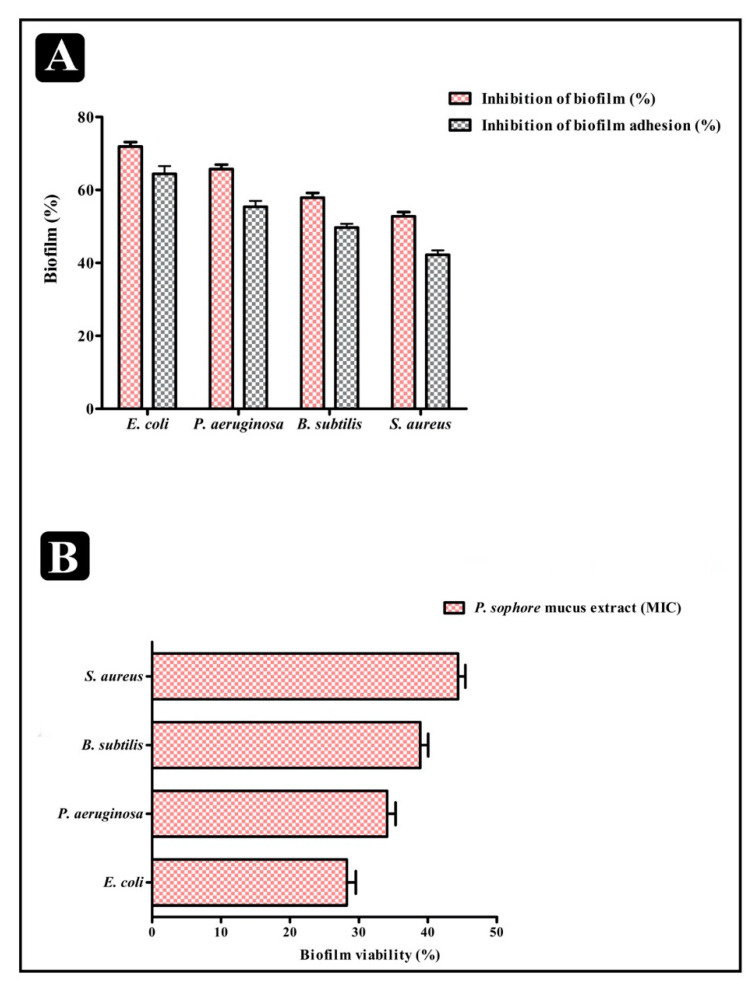
Antibiofilm potential of *P. sophore* mucus extract and XTT reduction assay. (**A**) Effect of *P. sophore* mucus extraction established biofilms and on adherence ability of *E. coli*, *P. aeruginosa, B. subtilis*, and *S. aureus* at their respective MICs. (**B**) Percentage of bacterial viability within biofilms measured by the XTT assay at respective MICs. All experiments were carried out in triplicate, and data represent the mean ± standard error of mean (SEM).

The antibiofilm ability of *P. sophore* mucus extract against four pathogenic bacteria was assayed by its ability to disrupt preformed biofilms and affecting their adhesion to surface. *Puntius sophore* mucus extract was capable enough to distort the preformed biofilms with an impact on their adhesion ability. Obtained results revealed that *P. sophore* mucus extract had an affinity to hinder the growth and preformed biofilms by hampering their adhesion potentiality at MIC. At this concentration, the inhibition of preformed biofilms by *P. sophore* mucus extract was about 71.91% for *E. coli*, 65.72% for *P. aeruginosa*, 57.87% for *B. subtilis*, and 52.77% for *S. aureus,* respectively. It was also found to decrease the adhesion ability of biofilms with percentage of inhibition as 64.42% for *E. coli*, 55.40% for *P. aeruginosa*, 49.67% for *B. subtilis*, and 42.26% for *S. aureus,* respectively (Figure 4A).

### 3.5. Effect of Puntius sophore Mucus Extract on Biofilms

To evaluate the effect of *P. sophore* mucus extract on biofilms of four pathogenic bacteria, we grew them in 96-well plates for 24 h. Non-adherent bacteria were then taken out and the mucus extract was added at respective MICs to treat the adherent bacteria for a further 24 h, followed by the XTT reduction assay to examine the viability of pathogenic bacteria within biofilms. As presented in Figure 4B, the viability of all bacteria within biofilms decreased significantly upon treatment with *P. sophore* mucus extract with different sensitivities.

### 3.6. Effect of Puntius sophore Mucus Extract on Bacterial Cells Entrapped in Biofilms

**Figure 5:**
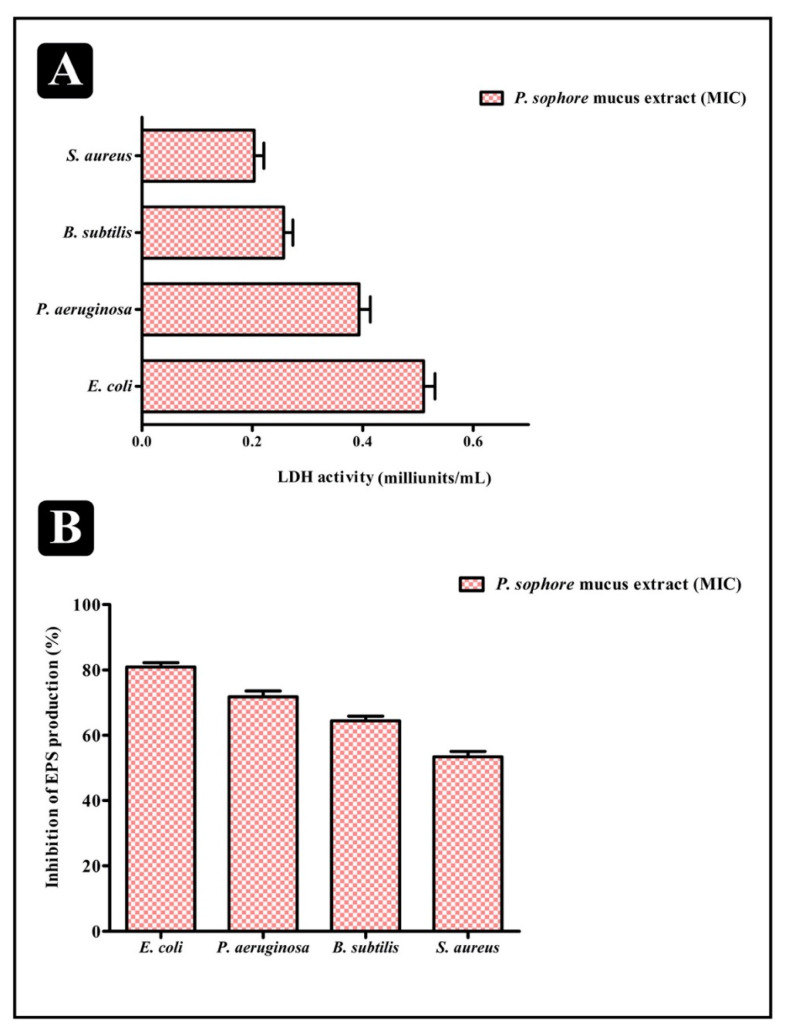
Result of LDH activity assay and total inhibition of EPS production (%). (**A**) Bacterial cell damage within the biofilm based on LDH activity in the presence of *P. sophore* mucus extract at their respective MICs. (**B**) Percentage inhibition of total EPS production in different bacterial strains in the presence of *P. sophore* mucus extract at their respective MICs. All experiments were carried out in triplicate, and data represent the mean ± SEM.

Bacterial intrinsic intracellular enzyme, lactate dehydrogenase (LDH) catalyzes the conversion of lactate to pyruvate and back. We evaluated LDH activity in the supernatant to check the probability that *P. sophore* mucus extract could reduce the bacterial viability inside the biofilms. When bacterial cell membrane is not intact, only then its activity can be detected in extracellular matrix. LDH activities in the supernatants are raised in all four bacteria upon the treatment of *P. sophore* mucus extract at the MIC level (Figure 5A). When comparing with the tested bacterial strains, highest LDH activity was seen in *E. coli*, while the *S. aureus* led to the lowest. These results demonstrate that the mucus extract of *P. sophore* could damage the cell membrane of bacteria within the biofilms, ultimately killing the bacteria. This can possibly be one mechanism for reducing biofilms by mucus extract. 

### 3.7. Extracellular Polysaccharide (EPS) Production

Inside the biofilms, bacterial cells produce EPS which aids in entrapping the nutrients. After the treatment of *P. sophore* mucus extract at MIC, total EPS production was remarkably decreased in all tested pathogens. In contrast to control, EPS production in *E. coli* and *P. aeruginosa* lowered by 80.91% and 71.73%, respectively; whereas in *B. subtilis* and *S. aureus,* it decreases by 64.39% and 53.42%*,* respectively (Figure 5B).

### 3.8. Visualization of Disrupted Biofilms by Microscopic Analysis (LM, FM, and SEM)

**Figure 6:**
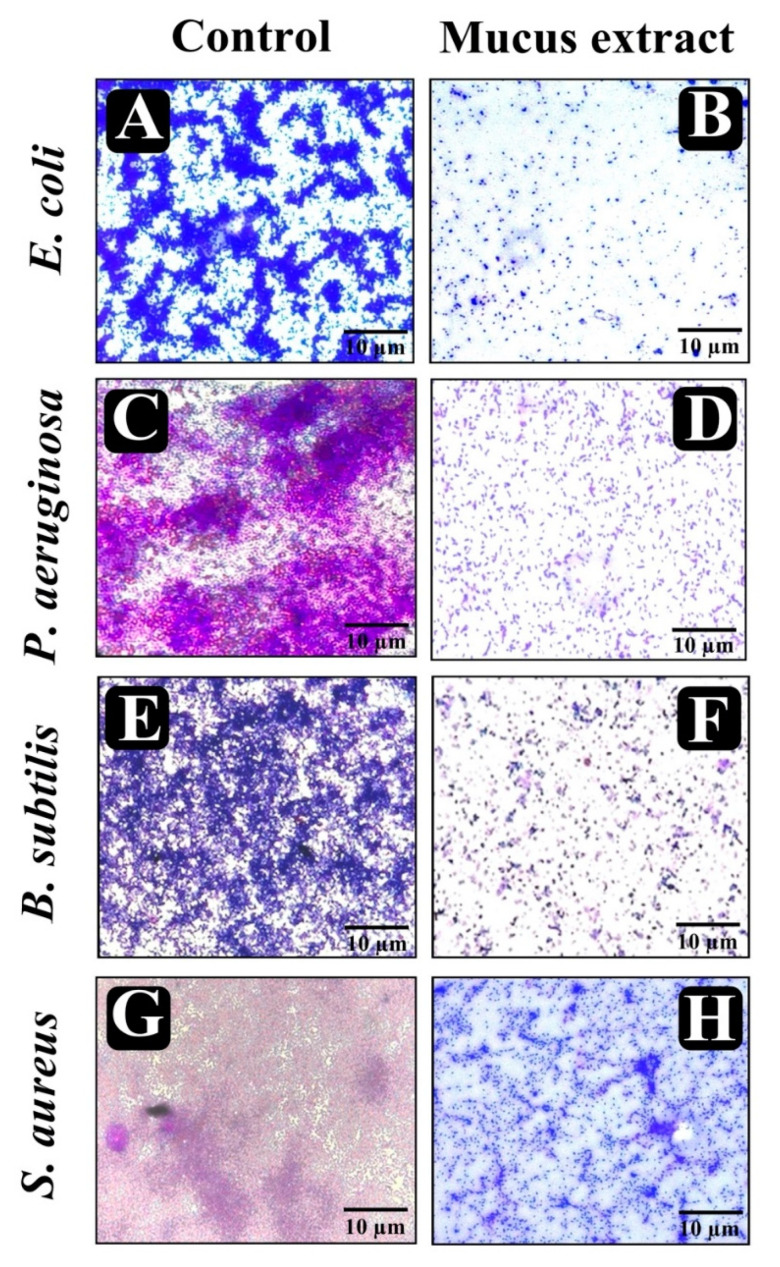
Micrographs of disrupted matured biofilms of tested strains formed on glass surfaces by *P. sophore* mucus extract at their respective MICs under light microscopy. (**A**,**C**,**E**,**G**) Growth control, (**B**,**D**,**F**,**H**) *P. sophore* mucus extract.

**Figure 7:**
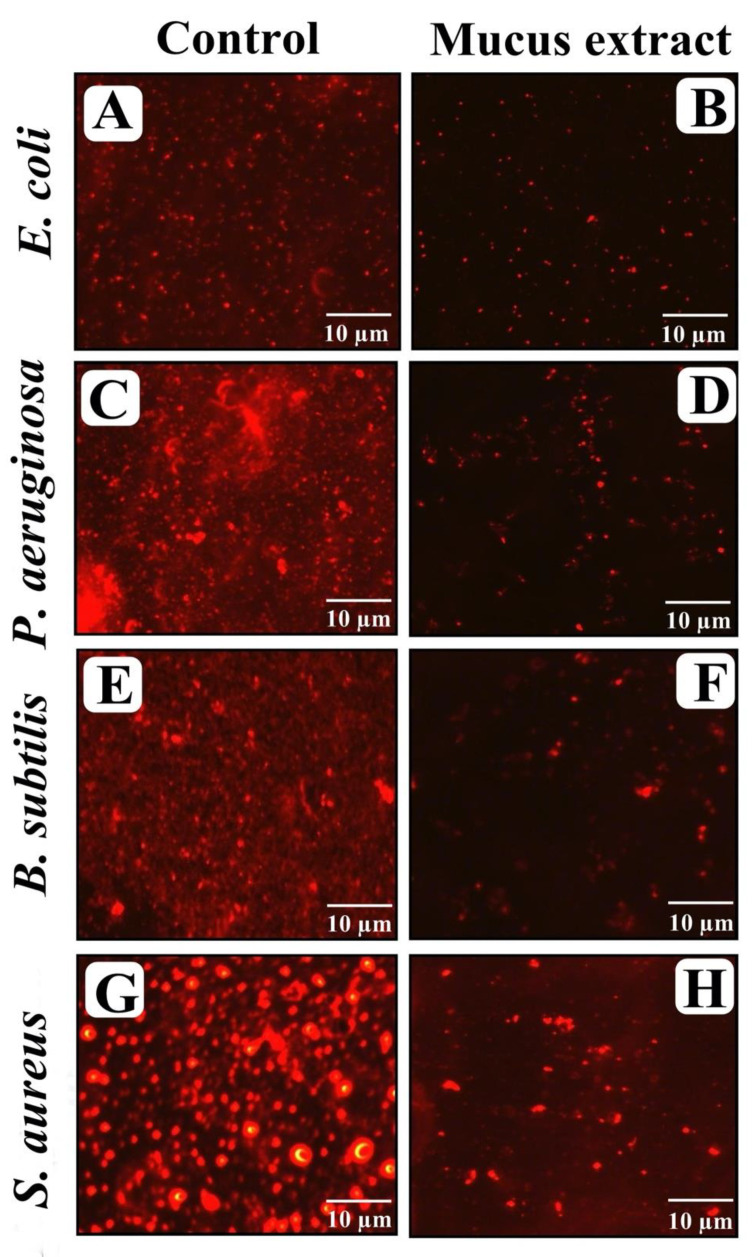
Micrographs of disrupted matured biofilms of tested strains formed on glass surfaces by *P. sophore* mucus extract at their respective MICs under fluorescent microscopy. (**A**,**C**,**E**,**G**) Growth control, (**B**,**D**,**F**,**H**) *P. sophore* mucus extract.

**Figure 8:**
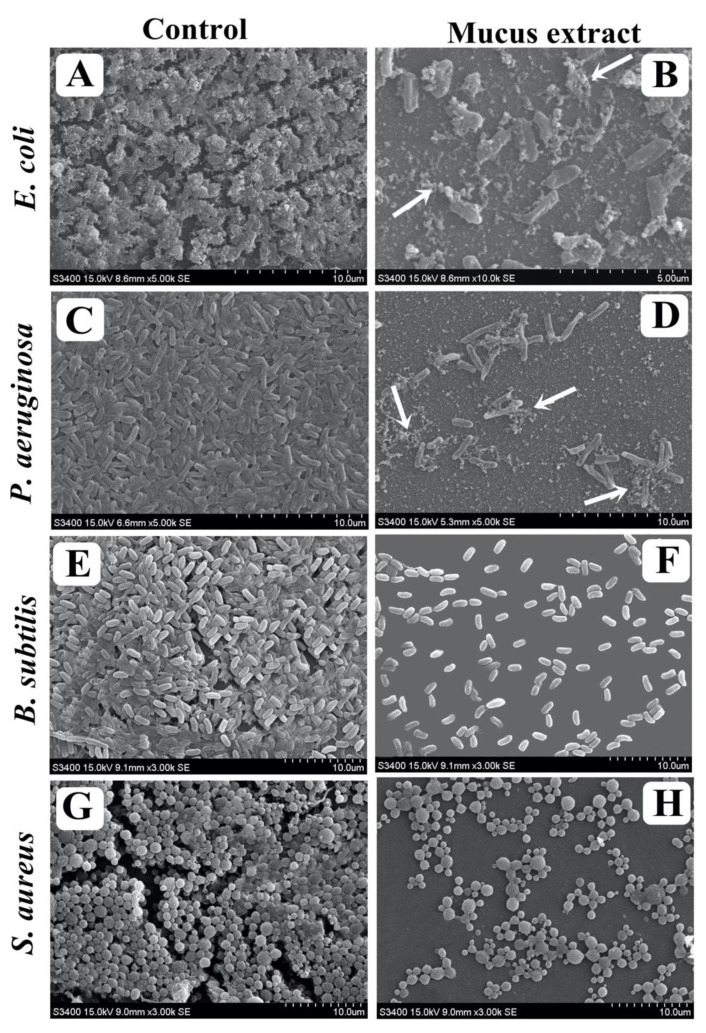
Micrographs of disrupted matured biofilms of tested strains formed on glass surfaces by *P. sophore* mucus extract at their respective MICs under scanning electron microscopy. (**A**,**C**,**E**,**G**) Growth control, (**B**,**D**,**F**,**H**) *P. sophore* mucus extract. Arrows indicated lysis of bacterial cells in (**B**) and (**D**).

The effect of *P. sophore* mucus extract at its MIC over matured biofilms developed on a glass surface that was stained with crystal violet and acridine orange to observe under light and fluorescence microscopy. In light microscopy, reductions in thickness of biofilm with lower appearance of micro colonies was observed in the presence of *P. sophore* mucus extract, when compared to control in which a heavy-knit like mat of biofilms appeared (Figure 6A–H). Moreover, results of fluorescence microscopy also revealed the well-developed mature biofilm in control, whereas bacterial strains treated with *P. sophore* mucus extract showed poor biofilm development (Figure 7A–H). In the second instance, SEM analysis was also performed to confirm the surface morphology and anatomy of biofilms formed by tested pathogens with or without *P. sophore* mucus extract. Prototypical multi-tiered growth of biofilms was observed in the control group, while the *P. sophore* mucus extract treated group displayed a reduction of thick aggregation of pathogenic bacteria compared to the control. This might be due to the degradation/reduction of the thick EPS layer present in the biofilms. This result was also complemented with the EPS assay in which EPS production was remarkably decreased in all pathogens treated with *P. sophore* mucus extract (Figure 8A–H). Thoroughly, our results have provided, altogether, evidence that *P. sophore* mucus extract has an effective antibiofilm potential against the different pathogens.

### 3.9. Cytotoxicity of Puntius sophore Mucus Extract to Normal Colon Cells

**Figure 9:**
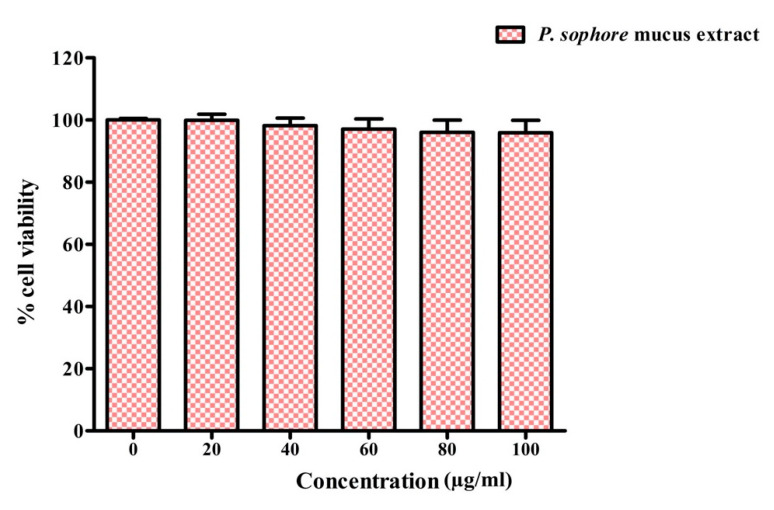
Cytotoxicity of *P. sophore* mucus extract on CRL-1831 cells. All experiments were carried out in triplicate, and data represent the mean ± SEM.

Finally, the cytotoxic effect of *P. sophore* mucus extract was also evaluated. The mucus extract showed no form of toxicity towards normal colon CRL-1831 cells. Viability of CRL-1831 cells were not altered after the treatment of *P. sophore* mucus extract (Figure 9). Therefore, our results indicated that *P. sophore* mucus extract inhibits the biofilm formation by pathogenic bacteria without any kind of cytotoxicity.

### 3.10. Bioactive Compounds Present in Puntius sophore Mucus Extract

**Figure 10:**
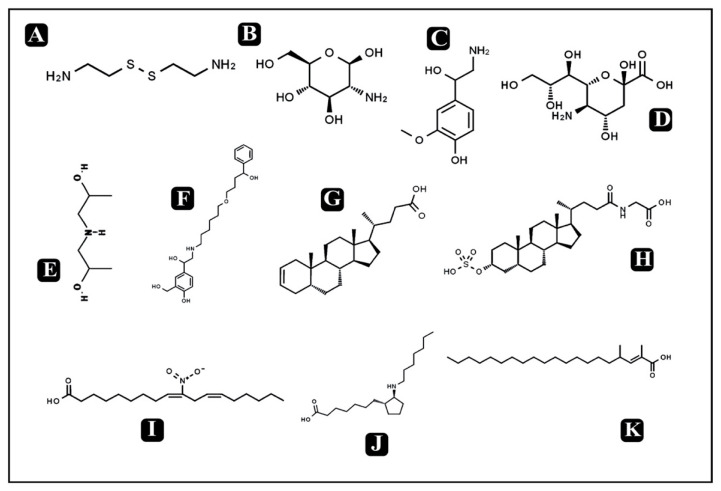
Chemical structures of identified compounds by HR-LCMS. (**A**) Cysteamine (**B**) glucosamine, (**C**) normetanephrine, (**D**) neuraminic acid, (**E**) bis (2-hydroxypropyl) amine, (**F**) hydroxysalmeterol, (**G**) 5-beta-chol-2-en-24-oic acid, (**H**) sulfolithocholylglycine, (**I**) 10-nitro,9Z,12Z-octadecadienoic acid, (**J**) 13-Azaprostanoic acid, (**K**) 2,4-dimethyl-2-eicosenoic acid.

**Figure 11:**
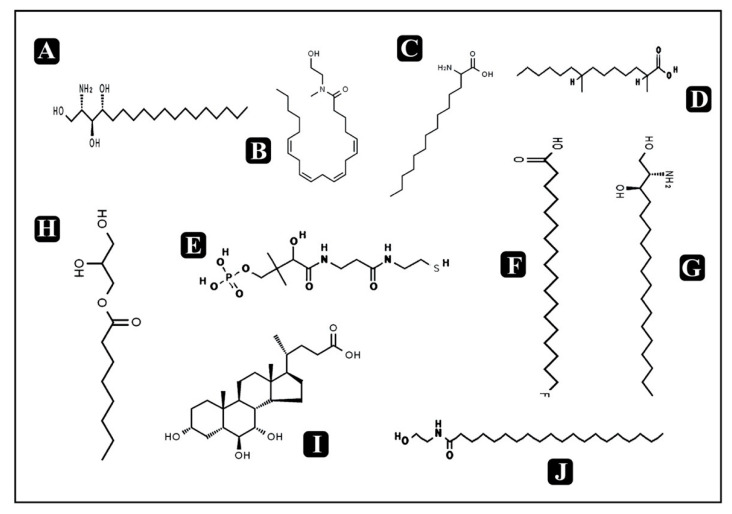
Chemical structures of identified compounds by HR-LCMS. (**A**) Phytosphingosine, (**B**) N-methyl *N*-(2-hydroxy-ethyl) arachidonoyl amine, (**C**) 2-amino-tetradecanoic acid, (**D**) 2,4-dimethyl-tetradecanoic acid (**E**) D-pantetheine 4’-phosphate, (**F**) 18-fluoro-octadecanoic acid, (**G**) dihydrosphingosine, (**H**) 1-octanoyl-rac-glycerol, (**I**) 3-alpha,6-beta,7-alpha-trihydroxy-5beta-cholan-24-oicacid, (**J**) *N*-(2-hydroxyethyl) icosanamide.

On the basis of significant antibacterial and antibiofilm potential, mucus extract of *P. sophore* was analyzed by HR-LCMS for determination of bioactive metabolites. They were putatively identified with their detailed mass spectra data, absorbance spectra, and retention times compared with human metabolome database. A large number of metabolites were detected from the skin mucus using both positive (+ESI) and negative electrospray (-ESI) ionization (Figure 10 and Figure 11). *P. sophore* mucus holds different classes of bioactive metabolites including fatty acids, lipids, amino sugars, amino alcohols, small peptides, etc. (Table 3).

## 4. Discussion

In recent years, one of the biggest matters of concern around the globe is fighting against the bacterial infections coupled with multidrug-resistance and biofilm forming. Bacteria are capable enough to grow and adhere to almost any kind of surface and develop architecturally complex communities termed as biofilms. Bacterial biofilms impact human beings in broad ways, as it can form in any natural, industrial, and medical setting [31,47,48]. Almost 65% of all bacterial infections are related to bacterial biofilms which includes both device (catheters, lenses, pacemakers, mechanical heart valves, etc.) and non-device related (periodontitis, tooth loss, osteomyelitis, etc.) infections [49]. Furthermore, bacteria inside biofilms are resistant to different antibiotics and any other chemical or environmental fluctuations compared to their planktonic form [50]. Therefore, all of these situations together with limitations of antibacterial drugs, encourages the advancement of novel remedial strategies to prevent bacterial biofilms and their related infections. 

Over the past decades, antimicrobial properties of natural products have been the center of attention of researchers for therapeutic innovations. Natural products are safe, as they are derived from natural resources [31,51,52] and do not affect the surfaces and surroundings while acting upon them. One such example is Hagfish, which is evolutionarily one of the most primitive species lacking vital and necessary adaptive defense mechanisms including antibody-based immunity and thymus, which are usually present in teleost fish [53,54]. However, still, they are known scavengers, inhabiting the ocean’s muddy bottom and survive in those conditions [55]. This suggests that, to survive in such conditions without defense components, they secrete a large amount of mucus comprised of effective antimicrobial compounds, which may possibly include bioactive peptides/proteins, lysozyme, and proteases [56]. Therefore, in the search of natural antibacterial and antibiofilm compounds, that are profoundly required to act on different biofilm forming pathogenic bacteria, we selected mucus extract of the fish *P. sophore*. The mucus extract of this medicinally important fish showed broad-spectrum antibacterial activity and was found to be enormously effective against both planktonic and biofilm forms of different pathogenic bacteria which are commonly involved in foodborne and healthcare associated human infections.

The aquatic environment is a habitat for numerous amounts of pathogenic and non-pathogenic microorganisms, and fish are everlastingly in connection with that surrounding. Fish epidermal mucus secretion and the epidermis itself functions as a biological barrier between the potential pathogens of its environment and fish [57]. Fish mucus is versatile, as it plays an important role in different activities, such as communication, respiration, feeding, reproduction, excretion, ionic and osmotic regulation, nest building, and resistance to diseases [58]. Many studies have demonstrated fish mucus as a potent source of novel antimicrobial compounds. It acts as a first line of defense against pathogens [59,60,61,62,63,64]. This was further proved in the present study as mucus extract of *P. sophore* presented a satisfactory antibacterial activity with MIC and MBC of about 250 and 500 µg/mL for *E. coli*, 500 and 1000 µg/mL for *P. aeruginosa*, 1000 and 1500 µg/mL for *B. subtilis*, 1500 and >1500 µg/mL for *S. aureus*, respectively (Table 2). Gram-negative strains were found to be more susceptible than Gram-positive strains, and this is due to the thickness and presence of the cell wall. Gram-positive bacteria possess a thick (20–80 nm) cell wall as the outer shell of the cell. In contrast, Gram-negative bacteria have a relatively thin (<10 nm) layer of cell wall, but have an additional outer membrane with several pores and appendices. These differences in the cell envelope confer different properties to the cell, in particular, response to the external stresses, including antimicrobial agents, heat, and UV radiation [65]. However, the main component of the cell wall is peptidoglycan, which is found in almost all bacteria and is responsible for preserving the integrity of the cell. Destruction of peptidoglycan either through mutations or external stresses (e.g., antibiotics) will lead to cell lysis [66,67]. Assessment of MIC and MBC are excellent and comparatively economical tools to concurrently assess many antimicrobial agents for effectiveness. Many studies have demonstrated similar results about the antimicrobial property of epidermal mucus in variety of fishes *Channa punctatus* and *Cirrhinusmrigala* [68], catfish (*Arius maculates*) [69], hagfish (*Myxine glutinosa*) [70], and eel fish (*Anguilla Anguilla*) [71]. Ellis [72] and Cole et al. [73] reported the variety of antimicrobial components (lysosomes, lecithin, proteases, and antimicrobial peptides) in the epidermal mucus. This bactericidal activity suggests that antimicrobial components present in the mucus extract play a key role in host defense against pathogenic infections.

Moreover, bacterial growth kinetics analysis was performed to evaluate the effect of mucus extract on growth of bacteria over time. The growth of all tested pathogenic bacteria in the presence of *P. sophore* mucus extract was indicated by delayed log phase and a slow logarithmic phase when compared to control. This time dependent killing of bacteria by *P. sophore* mucus extract indicated that the antibacterial activity could be because of the different cellular events like repression of macromolecular synthesis within the cell [74]. 

In view of antibacterial remedy, drug amalgamation has many advantages in comparison to the use of single agents. It may be employed to achieve synergistic activity, to impede emergence of resistant bacteria, and to lower the side effects because of the use of lower drug concentration [75]. The amalgamation of *P. sophore* mucus extract and gentamicin was imperative to optimize the antibacterial efficacy of both. Moreover, future studies are necessary for testing antibacterial resistance towards other drugs. *P. sophore* mucus extract also showed the remarkable results in inhibiting the biofilms of all tested bacterial pathogens in a concentration dependent manner at their respective MICs. The extract was also capable in distorting the preformed biofilms as well as obstructing the adhesion property of tested strains (Figure 5A,B). It also influences the viability of bacterial cells within biofilms. Results of XTT reduction assay indicated that the bacterial biofilms are decreased upon the treatment of *P. sophore* mucus extract (Figure 4B). Apart from this, mucus extract could also influence the bacterial integrity within the biofilm and damage it upon treatment, which possibly leads to the release of an intrinsic intracellular enzyme LDH (Figure 5A). A standard crystal violet and acridine orange assay intended for evaluating the biofilm biomass showed that *P. sophore* mucus extract was more effective in the extermination of preformed biofilms formed by all tested pathogens. This was further confirmed by SEM analysis by decreasing the multilayer growth of biofilms and free-living cells by influencing the integrity of cell wall. Additionally, it was also observed that disturbed cell walls of all bacterial strains led to failure in the emergence of clusters and inability to maintain their typical morphology in the presence of mucus extract. 

Moreover, extracellular polymeric substances (EPSs) produced by bacteria significantly contributes in their adhesion to the surface biofilm formation and structural integrity [76]. EPSs mediate the process of microcolony formation, leading to biofilm development. Therefore, EPS rich matrix with microcolonies is essential for physical stability, integrity, and attachment of biofilm to any surfaces [77]. Results of the present study revealed that *P. sophore* mucus extract carried out the inhibition of EPSs in all tested bacterial strains. Reduction in the biochemical constitution of the biofilm matrix weakens the complexity of biofilm and makes it easy for the drugs to access [78]. Altogether, our data demonstrated the same finding, that *P. sophore* mucus extract restricts the formation of biofilms.

Bioactive metabolites known to have antimicrobial potential and different classes of bioactive metabolites including fatty acids, lipids, amino sugars, amino alcohols, small peptides, etc., were identified from the *P. sophore* mucus via HR-LCMS analysis (Table 3). The detected fatty acids such as *13*-*azaprostanoic acid,* 2,4-dimethyl-2-eicosenoic acid, 2-amino-tetradecanoic acid, 10-nitro,9Z,12Z-octadecadienoic acid, 2,4-dimethyl-tetradecanoic acid, and 18-fluoro-octadecanoic acid could play an important role in antibacterial and antibiofilm potential of *P. sophore* mucus extract, as they have been found to have strong antibacterial activities via inhibiting different cellular activities like interfering with the bacterial membrane, enzyme activity inhibition oxidative phosphorylation uncoupling, auto-oxidation and peroxidation, disruption of electron transport chain, and via cell lysis [79]. It has been reported that free fatty acids are the major part of fish mucus and contribute in protection against a variety of fungal and bacterial diseases as human sebum [80]. Apart from this study, fatty acids are also detected in fish mucus samples of different species as the result of lipolysis of triglycerides [81,82]. 

Two other noteworthy metabolites, glucosamine and neuraminic acid, were also detected in the present study, which are associated with antimicrobial properties [40]. Moreover, our results also revealed the presence of two of the most important host-derived lipids; phytosphingosine and dihydrosphingosine, that have been known as antimicrobial molecules. They function in innate immune response along with peptides and are found on skin, saliva, and mucosal surfaces including other body fluids. Their possible mode of action is inhibition of cell wall synthesis and interfering with the bacterial membrane [44]. Another important detection in fish epidermal mucus is the occurrence of short peptides which are also known for antimicrobial and antibiofilm activities [14,83,84,85,86,87,88]. They are also known as host defense peptides and are a first line of defense against invading pathogens by providing direct (antimicrobial, antibiofilm,) or indirect (anti-inflammatory, immunomodulatory) defense against different microbial pathogens [89]. Moreover, recently discovered extracellular DNA (eDNA), which is also a biofilm component and observed in biofilms of specific bacteria like *P. aeruginosa* and *S. aureus*, also plays a crucial role in maintaining the integrity of biofilms [90]. Apart from the identified bioactive compounds from *P. sophore,* there is a possibility that DNases are also involved or partly functions in disintegrating the biofilm structure by degrading the eDNA from it. In order to further support this study, in vivo translation of obtained results should be performed. Identified bioactive compounds and peptides must be individually tested for efficacy and potency, which will represent a far more realistic prediction of every compound and peptide activity.

## 5. Conclusions

Collectively, this study revealed that *P. sophore* mucus contains a diverse class of bioactive metabolites that might have an exceptional antibacterial potential against all assessed Gram-positive and Gram-negative pathogenic bacteria. *P. sophore* mucus extract was found to inhibit biofilm formation by affecting the viability and integrity of bacterial cells within biofilms, as well as by hampering the production of EPS. These findings indicate *P. sophore* mucus can potentially be useful or can become a potent antibacterial and antibiofilm compound, as an alternative to antibiotics or other drug agents. Hence, we recommend more investigations to be conducted to have a better understanding about the broad action of mucus, before efforts are made to develop its pharmaceutical applications.

## Figures and Tables

**Table 1:** Antibacterial activity of *P. sophore* mucus extract.

Bacterial Strains	*P. sophore* Mucus Extract (µg/mL)		Gentamicin (µg/mL)	
	MIC	MBC	MIC	MBC
*E. coli*	250	500	7.8	15
*P. aeruginosa*	500	1000	15	31
*B. subtilis*	1000	1500	15	31
*S. aureus*	1500	>1500	15	31

where, MIC: Minimum Inhibitory Concentration. MBC: Minimum Bactericidal Concentration.

**Table 2:** FICI determination of *P. sophore* mucus extract.

Bacterial Strain	Mucus Extract	Gentamicin	FICI	Effect
	MIC *	MIC *		
*E. coli*	31	1.9	0.375	Synergy
*P. aeruginosa*	83	4.4	0.452	Synergy
*B. subtilis*	200	44	0.485	Synergy
*S. aureus*	375	44	0.535	Additive

* MIC in a combination of *P. sophore* mucus extract and Gentamicin (µg/mL). FICI evaluated as synergistic when FICI is <0.5; additive when the FICI is >0.5 to <2, and antagonistic when the FICI is >2. Where, MIC: Minimum Inhibitory Concentration. MBC: Minimum Bactericidal Concentration.

**Table 3:** Identified major bioactive metabolites by HR-LCMS from *P. sophore* mucus extract with their bioactivity.

Bioactive Metabolites	Formula	Class	*m/z*	RT (min)	Mass	Mode of Action	References
**Cysteamine**	C_4_H_12_N_2_S_2_	Organic disulfide	150.15	0.769	152.04	Anti-infective activity against bacteria, viruses, and malarial parasites	[38]
**Glucosamine**	C_6_H_13_NO_5_	Amino sugar	180.19	1.004	179.07	Antibacterial activity against different Gram- positive and Gram-negative bacteria and antifungal activity against few fungal strains	[39]
**Lys Ser Phe**	C_18_H_28_N_4_O_5_	Small peptide	387.46	1.026	380.20	-	
**Met Lys**	C_11_H_23_N_3_O_3_S	Small peptide	282.59	1.034	277.14	-	
**Normetanephrine**	C_9_H_13_NO_3_	Catecholamine	181.07	1.046	183.08	-	
**Dodecaprenylphosphategalacturonic acid**	C_31_H_51_O_10_P	Bactoprenol	620.64	1.048	614.32	-	
**Bis (2-hydroxypropyl) amine**	C_6_H_15_NO_2_	Amino alcohol	130.24	1.096	133.11	-	
**Lys Met Thr**	C_15_H_30_N_4_O_5_S	Small peptide	380.20	1.1	378.19	-	
**Neuraminic acid**	C_9_H_17_NO_8_	Amino sugar	272.67	1.443	267.09	Antibacterial activity against different Gram- positive and Gram-negative bacteria and antifungal activity against few fungal strains	[40]
**Thr Ile Tyr**	C_19_H_29_N_3_O_6_	Small peptide	403.27	3.217	395.20	-	
**Pro ArgGln**	C_16_H_29_N_7_O_5_	Small peptide	398.26	4.654	399.22	-	
**Hydroxysalmeterol**	C_25_H_37_NO_5_	Amino alcohol	433.29	4.836	431.27	-	
**5beta-Chol-2-en-24-oic Acid**	C_24_H_38_O_2_	Alcohol	356.46	6.578	358.28	Antimicrobialactivity	[41]
**Sulfolithocholylglycine**	C_26_H_43_NO_7_S	Glycine conjugate (sterol lipid)	512.29	7.034	513.27	-	
**10-nitro,9Z,12Z-octadecadienoic acid**	C_18_H_31_NO_4_	Fatty acids	325.20	7.59	325.22	Antimicrobial activity against oral pathogens	[42]
**3-Ketosphingosine**	C_18_H_35_NO_2_	Sphingosine (sphingolipid)	296.24	9.879	297.26	-	
**GlnGln Met**	C_15_H_27_N_5_O_6_S	Small peptide	404.19	10.039	405.17	-	
**13-Azaprostanoic acid**	C_19_H_37_NO_2_	Fatty acids	311.20	10.51	311.28	Gastroprotective activity	
**2,4-Dimethyl-2-eicosenoic acid**	C_22_H_42_O_2_	Fatty acids	337.32	10.841	338.31		
**Phytosphingosine**	C_18_H_39_NO_3_	Sphingolipids	315.31	11.247	317.29	Anti-inflammatory and antimicrobial activity against different bacteria and yeast	[43]
**N-methyl N-(2-hydroxy-ethyl) arachidonoyl amine**	C_23_H_39_NO_2_	Fatty amides	360.16	11.691	361.29	-	
**ArgGlnPhe**	C_20_H_31_N_7_O_5_	Small peptide	447.35	11.958	449.23	-	
**2-Amino-tetradecanoic acid**	C_14_H_29_NO_2_	Fatty acids	244.20	12.626	243.21		
**2,4-Dimethyl-tetradecanoic acid**	C_16_H_32_O_2_	Fatty acids	256.23	13.833	256.24		
**D-Pantetheine 4’-phosphate**	C_11_H_23_N_2_O_7_PS	Organophosphorus compound	356.09	14.121	358.09	-	
**18-Fluoro-octadecanoic acid**	C_18_H_35_FO_2_	Fatty acids	302.19	18.385	302.26		
**Dihydrosphingosine**	C_18_H_39_NO_2_	Amino alcohol	309.35	18.774	301.29	Antimicrobial activity against a variety of opportunistic bacteria, viruses and fungi	[44]
**1-Octanoyl-rac-glycerol**	C_11_H_22_O_4_	Monoacylglyc-erol (glycerolipid)	220.31	18.875	218.29	Antibacterial activity against *D. congolensis*, *Campylobacter* spp., *E. coli*, *Listeria* spp., and *Salmonella* spp.	[45]
**GlnArg Lys**	C_17_H_34_N_8_O_5_	Small peptide	437.45	19.598	430.26	-	
**3alpha,6beta,7alpha-Trihydroxy-5beta-cholan-24-oic acid**	C_24_H_40_O_5_	Sterol lipid	410.29	19.622	408.28	Antibacterial activity against *Bifidobacterium breve, Blautiacoccoides,* and *Bacteroides thetaiotaomicron*	[46]
**PheGlnArg**	C_20_H_31_N_7_O_5_	Small peptide	457.21	19.879	449.23	-	
**N-(2-hydroxyethyl) icosanamide**	C_22_H_45_NO_2_	Endocannabinoids	360.37	20.094	355.34	-	
**Lys Gln Leu**	C_17_H_33_N_5_O_5_	Small peptide	397.23	26.95	387.24	-	
**Arg Ser Ser**	C_12_H_24_N_6_O_6_	Small peptide	352.20	26.995	348.17	-	
**ArgGlnArg**	C_17_H_34_N_10_O_5_	Small peptide	462.11	26.997	458.26	-	
**Ala Lys Ile**	C_15_H_30_N_4_O_4_	Small peptide	349.28	27.063	330.22	-	
**Ile Thr Pro**	C_15_H_27_N_3_O_5_	Small peptide	355.39	27.072	329.19	-	
**Lys Gln Leu**	C_17_H_33_N_5_O_5_	Small peptide	390.21	27.118	387.24	-	

## References

[1] Donlan R.M. (2002). Biofilms: Microbial Life on Surfaces. Emerg. Infect. Dis..

[2] Limoli D., Jones C.J., Wozniak D.J. (2015). Bacterial Extracellular Polysaccharides in Biofilm Formation and Function. Microbiol. Spectr..

[3] Karygianni L., Ren Z., Koo H., Thurnheer T. (2020). Biofilm Matrixome: Extracellular Components in Structured Microbial Communities. Trends Microbiol..

[4] Flemming H.C. (2016). EPS-Then and Now. Microorganisms.

[5] Singh S., Singh S.K., Chowdhury I., Singh R. (2017). Understanding the Mechanism of Bacterial Biofilms Resistance to Antimicrobial Agents. Open Microbiol. J..

[6] Ciofu O., Tolker-Nielsen T. (2019). Tolerance and Resistance of Pseudomonas aeruginosa Biofilms to Antimicrobial Agents-How P. aeruginosa Can Escape Antibiotics. Front. Microbiol..

[7] Sharma D., Misba L., Khan A.U. (2019). Antibiotics versus biofilm: An emerging battleground in microbial communities. Antimicrob. Resist. Infect. Control..

[8] Lebeaux D., Ghigo J.-M., Beloin C. (2014). Biofilm-Related Infections: Bridging the Gap between Clinical Management and Fundamental Aspects of Recalcitrance toward Antibiotics. Microbiol. Mol. Biol. Rev..

[9] Wu H., Moser C., Wang H.-Z., Høiby N., Song Z.-J. (2015). Strategies for combating bacterial biofilm infections. Int. J. Oral Sci..

[10] Jakobsen T.H., Tolker-Nielsen T., Givskov M. (2017). Bacterial Biofilm Control by Perturbation of Bacterial Signaling Processes. Int. J. Mol. Sci..

[11] Adnan M., Alshammari E., Patel M., Ashraf S.A., Khan S., Hadi S. (2018). Significance and potential of marine microbial natural bioactive compounds against biofilms/biofouling: Necessity for green chemistry. PeerJ.

[12] Azad C.S., Saxena M., Siddiqui A.J., Bhardwaj J., Puri S.K., Dutta G.P., Anand N., Saxena A.K., Anand N. (2017). Synthesis of primaquine glyco-conjugates as potential tissue schizontocidal antimalarial agents. Chem. Biol. Drug Des..

[13] Adnan M. (2019). Bioactive potential of essential oil extracted from the leaves of *Eucalyptus globulus* (Myrtaceae). J. Pharmacogn. Phytochem..

[14] Adnan M., Patel M., Deshpande S., Alreshidi M., Siddiqui A.J., Reddy M.N., Emira N., De Feo V. (2020). Effect of Adiantum philippense Extract on Biofilm Formation, Adhesion With Its Antibacterial Activities Against Foodborne Pathogens, and Characterization of Bioactive Metabolites: An in vitro-in silico Approach. Front. Microbiol..

[15] Alshammari E., Patel M., Sachidanandan M., Kumar P., Adnan M. (2019). Potential Evaluation and Health Fostering Intrinsic Traits of Novel Probiotic Strain Enterococcus durans F3 Isolated from the Gut of Fresh Water Fish Catla catla. Food Sci. Anim. Resour..

[16] Mahanty A., Ganguly S., Verma A., Sahoo S., Mitra P., Paria P., Sharma A.P., Singh B.K., Mohanty B.P. (2014). Nutrient Profile of Small Indigenous Fish Puntius sophore: Proximate Composition, Amino Acid, Fatty Acid and Micronutrient Profiles. Natl. Acad. Sci. Lett..

[17] Ahamed F., Ahmed Z.F., Hossain M.Y., Ohtomi J. (2012). Growth study of the Pool *Barb Puntius sophore* (Cyprinidae: Barbinae) through multi model inferences. Zool. Stud..

[18] Sarjubala W.M.S., Hawaibam R., Chungkham S. (2018). Nutritional properties of some freshwater fish species of Manipur, India. J. Coldwater Fish..

[19] Adnan M., Patel M., Reddy M.N., Alshammari E. (2018). Formulation, evaluation and bioactive potential of Xylaria primorskensis terpenoid nanoparticles from its major compound xylaranic acid. Sci. Rep..

[20] Adnan M., Patel M., Hadi S. (2017). Functional and health promoting inherent attributes of Enterococcus hirae F2 as a novel probiotic isolated from the digestive tract of the freshwater fish Catla catla. PeerJ.

[21] Diamond G., Zasloff M., Eck H., Brasseur M., Maloy W.L., Bevins C.L. (1991). Tracheal antimicrobial peptide, a cysteine-rich peptide from mammalian tracheal mucosa: Peptide isolation and cloning of a cDNA. Proc. Natl. Acad. Sci. USA.

[22] Clinical and Laboratory Standards Institute (CLSI) (2014). Performance Standards for Antimicrobial Susceptibility Testing. Twenty Fourth Informational Supplement, M100-S24..

[23] Kuete V. (2017). African Medicinal Spices and Vegetables and Their Potential in the Management of Metabolic Syndrome. Medicinal Spices and Vegetables from Africa.

[24] Silveira C.P., Torres-Rodriguez J.M., Alvarado-Ramírez E., Murciano-Gonzalo F., Dolande M., Panizo M., Reviakina V. (2009). MICs and minimum fungicidal concentrations of amphotericin B, itraconazole, posaconazole and terbinafine in Sporothrix schenckii. J. Med. Microbiol..

[25] Pillai S.K., Moellering R.C., Eliopoulos G.M., Lorian V. (2005). Antimicrobial combinations. Antibiotics in Laboratory Medicine.

[26] Božić D.D., Milenković M., Ivković B., Ćirković I. (2014). Antibacterial activity of three newly-synthesized chalcones & synergism with antibiotics against clinical isolates of methicillin-resistant Staphylococcus aureus. Indian J. Med. Res..

[27] Lee K.W.K., Periasamy S., Mukherjee M., Xie C., Kjelleberg S., Rice S.A. (2013). Biofilm development and enhanced stress resistance of a model, mixed-species community biofilm. ISME J..

[28] Nostro A., Roccaro A.S., Bisignano G., Marino A., Cannatelli M.A., Pizzimenti F.C., Cioni P.L., Procopio F., Blanco A.R. (2007). Effects of oregano, carvacrol and thymol on Staphylococcus aureus and Staphylococcus epidermidis biofilms. J. Med. Microbiol..

[29] Plyuta V., Zaitseva J., Lobakova E., Zagoskina N., Kuznetsov A., Khmel I.A. (2013). Effect of plant phenolic compounds on biofilm formation byPseudomonas aeruginosa. APMIS.

[30] Musthafa K.S., Ravi A.V., Annapoorani A., Packiavathy S.V., Pandian S.K. (2010). Evaluation of Anti-Quorum-Sensing Activity of Edible Plants and Fruits through Inhibition of the N-Acyl-Homoserine Lactone System in Chromobacterium violaceum and Pseudomonas aeruginosa. Chemotherapy.

[31] Adnan M., Sousa A.M., Machado I., Pereira M.O., Khan S., Morton G., Hadi S. (2017). Role of bolA and rpoS genes in biofilm formation and adherence pattern by Escherichia coli K-12 MG1655 on polypropylene, stainless steel, and silicone surfaces. Acta Microbiol. Immunol. Hung..

[32] Patel M., Reddy M.N. (2018). Discovery of the World’s Smallest Terrestrial Pteridophyte. Sci. Rep..

[33] Ramage G., Walle K.V., Wickes B.L., López-Ribot J.L. (2001). Standardized Method for In Vitro Antifungal Susceptibility Testing of Candida albicansBiofilms. Antimicrob. Agents Chemother..

[34] Nett J.E., Cain M.T., Crawford K., Andes D.R. (2011). Optimizing a Candida Biofilm Microtiter Plate Model for Measurement of Antifungal Susceptibility by Tetrazolium Salt Assay. J. Clin. Microbiol..

[35] Siddiqui A.J., Bhardwaj J., Goyal M., Prakash K., Adnan M., Alreshidi M.M., Patel M., Soni A., Redman W. (2020). Immune responses in liver and spleen against Plasmodium yoelii pre-erythrocytic stages in Swiss mice model. J. Adv. Res..

[36] Borucki M.K., Krug M.J., Muraoka W.T., Call D.R. (2003). Discrimination among Listeria monocytogenes isolates using a mixed genome DNA microarray. Veter. Microbiol..

[37] Reddy M.N., Adnan M., Alreshidi M.M., Saeed M., Patel M. (2020). Evaluation of Anticancer, Antibacterial and Antioxidant Properties of a Medicinally Treasured Fern Tectaria coadunata with its Phytoconstituents Analysis by HR-LCMS. Anti-Cancer Agents Med. Chem..

[38] Fraser-Pitt D.J., Mercer D.K., Smith D., Kowalczuk A., Robertson J., Lovie E., Perenyi P., Cole M.J., Doumith M., Hill R.L.R. (2018). Cysteamine, an Endogenous Aminothiol, and Cystamine, the Disulfide Product of Oxidation, IncreasePseudomonas aeruginosaSensitivity to Reactive Oxygen and Nitrogen Species and Potentiate Therapeutic Antibiotics against Bacterial Infection. Infect. Immun..

[39] Malik S., Singh M., Mathur A. (2013). Antimicrobial Activity of Food Grade Glucosamine. Int. J. Biotechnol. Bioeng. Res..

[40] Sakko M., Moore C., Novak-Frazer L., Rautemaa V., Sorsa T., Hietala P., Järvinen A., Bowyer P., Tjäderhane L., Rautemaa R. (2013). 2-hydroxyisocaproic acid is fungicidal for CandidaandAspergillusspecies. Mycoses.

[41] Bellini A.M., Mencini E., Quaglio M.P., Guameri M., Fini A. (1991). Antimicrobial activity of basic cholane derivatives. X. Synthesis of 3α- and 3β-amino-5β-cholan-24-oic acids. Steroids.

[42] Choi J.-S., Park N.-H., Hwang S.-Y., Sohn J.H., Kwak I., Cho K.K., Choi I.S. (2013). The antibacterial activity of various saturated and unsaturated fatty acids against several oral pathogens. J. Environ. Biol..

[43] Başpınar Y., Kotmakçı M., Öztürk I. (2018). Antimicrobial Activity of Phytosphingosine Nanoemulsions against Bacteria and Yeasts. Celal Bayar Üniv. Fen Bilimleri Derg..

[44] Fischer C.L. (2020). Antimicrobial Activity of Host-Derived Lipids. Antibiotics.

[45] Chang S.-S., Redondo-Solano M., Thippareddi H. (2010). Inactivation of Escherichia coli O157:H7 and Salmonella spp. on alfalfa seeds by caprylic acid and monocaprylin. Int. J. Food Microbiol..

[46] Watanabe M., Fukiya S., Yokota A. (2017). Comprehensive evaluation of the bactericidal activities of free bile acids in the large intestine of humans and rodents. J. Lipid Res..

[47] Adnan M., Morton G., Hadi S. (2011). Analysis of rpoS and bolA gene expression under various stress-induced environments in planktonic and biofilm phase using 2−ΔΔCT method. Mol. Cell. Biochem..

[48] Adnan M., Morton G., Singh J., Hadi S. (2010). Contribution of rpoS and bolA genes in biofilm formation in Escherichia coli K-12 MG1655. Mol. Cell. Biochem..

[49] Jamal M., Ahmad W., Andleeb S., Jalil F., Imran M., Nawaz M.A., Hussain T., Ali M., Rafiq M., Kamil M.A. (2018). Bacterial biofilm and associated infections. J. Chin. Med. Assoc..

[50] Song F., Koo H., Ren D. (2015). Effects of Material Properties on Bacterial Adhesion and Biofilm Formation. J. Dent. Res..

[51] Adnan M., Alshammari E., Ashraf S.A., Patel K., Lad K., Patel M. (2018). Physiological and Molecular Characterization of Biosurfactant Producing Endophytic Fungi Xylaria regalis from the Cones of Thuja plicata as a Potent Plant Growth Promoter with Its Potential Application. BioMed Res. Int..

[52] Adnan M., Ashraf S.A., Khan S., Alshammari E., AwadElkareem A.M. (2017). Effect of pH, temperature and incubation time on cordycepin production from Cordyceps militaris using solid-state fermentation on various substrates. CyTA - J. Food.

[53] Raison R.L., Dos Remedios N. (1998). The Hagfish Immune System. The Biology of Hagfishes.

[54] Rolff J. (2007). Why did the acquired immune system of vertebrates evolve?. Dev. Comp. Immunol..

[55] Spitzer R.H., Koch E.A. (1998). Hagfish Skin and Slime Glands. The Biology of Hagfishes.

[56] Subramanian S., MacKinnon S.L., Ross N.W. (2007). A comparative study on innate immune parameters in the epidermal mucus of various fish species. Comp. Biochem. Physiol. Part B Biochem. Mol. Biol..

[57] Shephard K.L. (1993). Mucus on the epidermis of fish and its influence on drug delivery. Adv. Drug Deliv. Rev..

[58] Button B., Boucher R.C., University of North Carolina Virtual Lung Group (2008). Role of mechanical stress in regulating airway surface hydration and mucus clearance rates. Respir. Physiol. Neurobiol..

[59] Austin B., McIntosh D. (1988). Natural antibacterial compounds on the surface of rainbow trout, Salmo gairdneri Richardson. J. Fish Dis..

[60] Fouz B., Devesa S., Gravningen K., Barja J.L., Toranzo A.E. (1990). Antibacterial action of the mucus of turbot. Bull. Eur. Assoc. Fish Pathol..

[61] Hjelmeland K., Christie M., Raa J. (1983). Skin mucus protease from rainbow trout, Salmo gairdneri Richardson, and its biological significance. J. Fish Biol..

[62] Grinde B., Jollès J., Jollès P. (1988). Purification and characterization of two lysozymes from rainbow trout (Salmo gairdneri). JBIC J. Biol. Inorg. Chem..

[63] Nagashima Y., Sendo A., Shimakura K., Shiomi K., Kobayashi T., Kimura B., Fujii T. (2001). Antibacterial factors in skin mucus of rabbitfishes. J. Fish Biol..

[64] Sarmaşik A. (2002). Antimicrobial peptides: A potential therapeutic alternative for the treatment of fish diseases. Turk. J. Biol..

[65] Mai-Prochnow A., Clauson M., Hong J., Murphy A.B. (2016). Gram positive and Gram negative bacteria differ in their sensitivity to cold plasma. Sci. Rep..

[66] Rogers H.J., Perkins H.R., Ward J.B. (1980). Microbial Cell Walls and Membranes. Microb. Cell Walls Membr..

[67] Vollmer W., Blanot D., De Pedro M.A. (2008). Peptidoglycan structure and architecture. FEMS Microbiol. Rev..

[68] Kuppulakshmi C., Prakash M., Gunasekaran G., Manimegalai G., Sarojini S. (2008). Antibacterial properties of fish mucus from Channa punctatus and Cirrhinus mrigala. Eur. Rev. Med. Pharmacol. Sci..

[69] Manivasagan P., Neelamegam A., Ashokkumar S., Palanisamy S. (2009). Studies on the proteinaceous gel secretion from the skin of the catfish, *Arius maculatus* (Thunberg, 1792). Afr. J. Biotechnol..

[70] Subramanian S., Ross N.W., MacKinnon S.L. (2008). Comparison of antimicrobial activity in the epidermal mucus extracts of fish. Comp. Biochem. Physiol. Part B Biochem. Mol. Biol..

[71] Bragadeeswaran S., Thangaraj S. (2011). Hemolytic and Antibacterial Studies on Skin Mucus of Eel Fish, Anguilla anguilla Linnaues, 1758. Asian J. Biol. Sci..

[72] Ellis A. (1999). Immunity to bacteria in fish. Fish Shellfish. Immunol..

[73] Cole A.M., Weis P., Diamond G. (1997). Isolation and Characterization of Pleurocidin, an Antimicrobial Peptide in the Skin Secretions of Winter Flounder. J. Biol. Chem..

[74] Saritha K., Rajesh A., Manjulatha K., Setty O.H., Yenugu S. (2015). Mechanism of antibacterial action of the alcoholic extracts of Hemidesmus indicus (L.) R. Br. ex Schult, Leucas aspera (Wild.), Plumbago zeylanica L., and Tridax procumbens (L.) R. Br. ex Schult. Front. Microbiol..

[75] Ocampo P.S., Lázár V., Papp B., Arnoldini M., Wiesch P.A.Z., Busa-Fekete R., Fekete G., Pál C., Ackermann M., Bonhoeffer S. (2014). Antagonism between Bacteriostatic and Bactericidal Antibiotics Is Prevalent. Antimicrob. Agents Chemother..

[76] Leme A.P., Koo H., Bellato C., Bedi G., Cury J. (2006). The Role of Sucrose in Cariogenic Dental Biofilm Formation—New Insight. J. Dent. Res..

[77] Kim N., Hwang G., Liu Y., Wang Y., Singh A.P., Vorsa N., Koo H. (2015). Cranberry Flavonoids Modulate Cariogenic Properties of Mixed-Species Biofilm through Exopolysaccharides-Matrix Disruption. PLoS ONE.

[78] Lentino J.R. (2003). Prosthetic Joint Infections: Bane of Orthopedists, Challenge for Infectious Disease Specialists. Clin. Infect. Dis..

[79] Desbois A., Smith V.J. (2009). Antibacterial free fatty acids: Activities, mechanisms of action and biotechnological potential. Appl. Microbiol. Biotechnol..

[80] Lewis R.W. (1970). Fish cutaneous mucus: A new source of skin surface lipid. Lipids.

[81] Ekman D.R., Skelton D.M., Davis J.M., Villeneuve D.L., Cavallin J.E., Schroeder A., Jensen K.M., Ankley G.T., Collette T.W. (2015). Metabolite Profiling of Fish Skin Mucus: A Novel Approach for Minimally-Invasive Environmental Exposure Monitoring and Surveillance. Environ. Sci. Technol..

[82] Jais A.M.M., Matori M., Kittakoop P., Sowanborirux K. (1998). Fatty Acid Compositions in Mucus and Roe of Haruan, Channa Striatus, for Wound Healing. Gen. Pharmacol. Vasc. Syst..

[83] Di Somma A., Moretta A., Canè C., Cirillo A., Duilio A. (2020). Antimicrobial and Antibiofilm Peptides. Biomolecules.

[84] Galdiero E., Lombardi L., Falanga A., Libralato G., Guida M., Carotenuto R. (2019). Biofilms: Novel Strategies Based on Antimicrobial Peptides. Pharmaceutics.

[85] Kim M.K., Kang H.K., Ko S.J., Hong M.J., Bang J.K., Seo C.H., Park Y. (2018). Mechanisms driving the antibacterial and antibiofilm properties of Hp1404 and its analogue peptides against multidrug-resistant Pseudomonas aeruginosa. Sci. Rep..

[86] Chung P.Y., Khanum R. (2017). Antimicrobial peptides as potential anti-biofilm agents against multidrug-resistant bacteria. J. Microbiol. Immunol. Infect..

[87] Andrea A., Molchanova N., Jenssen H. (2018). Antibiofilm Peptides and Peptidomimetics with Focus on Surface Immobilization. Biomolecules.

[88] Raheem N., Straus S.K. (2019). Mechanisms of Action for Antimicrobial Peptides with Antibacterial and Antibiofilm Functions. Front. Microbiol..

[89] Dostert M., Belanger C.R., Hancock R.E. (2018). Design and Assessment of Anti-Biofilm Peptides: Steps toward Clinical Application. J. Innate Immun..

[90] Montanaro L., Poggi A., Visai L., Ravaioli S., Campoccia D., Speziale P., Arciola C.R. (2011). Extracellular DNA in Biofilms. Int. J. Artif. Organs.

